# Exosomal miR-21-5p derived from gastric cancer promotes peritoneal metastasis via mesothelial-to-mesenchymal transition

**DOI:** 10.1038/s41419-018-0928-8

**Published:** 2018-08-28

**Authors:** Qiang Li, Bowen Li, Qing Li, Song Wei, Zhongyuan He, Xiaoxu Huang, Lu Wang, Yiwen Xia, Zhipeng Xu, Zheng Li, Weizhi Wang, Li Yang, Diancai Zhang, Zekuan Xu

**Affiliations:** Department of General Surgery, The First Affiliated Hospital of Nanjing Medical University, Nanjing, 210029 Jiangsu province China

## Abstract

Peritoneal metastasis is a primary metastatic route for gastric cancers, and the mechanisms underlying this process are still unclear. Peritoneal mesothelial cells (PMCs) undergo mesothelial-to-mesenchymal transition (MMT) to provide a favorable environment for metastatic cancer cells. In this study, we investigated how the exosomal miR-21-5p induces MMT and promotes peritoneal metastasis. Gastric cancer (GC)-derived exosomes were identified by transmission electron microscopy and western blot analysis, then the uptake of exosomes was confirmed by PKH-67 staining. The expression of miR-21-5p and SMAD7 were measured by quantitative real-time polymerase chain reaction (qRT-PCR) and western blot, and the interactions between miR-21-5p and its target genes SMAD7 were confirmed by Luciferase reporter assays. The MMT of PMCs was determined by invasion assays, adhesion assays, immunofluorescent assay, and western blot. Meanwhile, mouse model of tumor peritoneal dissemination model was performed to investigate the role of exosomal miR-21-5p in peritoneal metastasis in vivo. We found that PMCs could internalize GC-derived exosomal miR-21-5p and led to increased levels of miR-21-5p in PMCs. Through various types of in vitro and in vivo assays, we confirmed that exosomal miR-21-5p was able to induce MMT of PMCs and promote tumor peritoneal metastasis. Moreover, our study revealed that this process was promoted by exosomal miR-21-5p through activating TGF-β/Smad pathway via targeting SMAD7. Altogether, our data suggest that exosomal miR-21-5p induces MMT of PMCs and promote cancer peritoneal dissemination by targeting SMAD7. The exosomal miR-21-5p may be a novel therapeutic target for GC peritoneal metastasis.

## Introduction

Gastric cancer (GC) is one of the most common cancers worldwide, with more than 50% of cases occurring in Eastern Asia^1^. In china, GC has become the second leading cause of cancer deaths^2^. According to the national survey, the number of new GC cases in China in 2015 was 679,000, with 498,000 deaths. Although surgery, radiotherapy, chemotherapy and biological treatment have been adopted so far, the 5-year survival rate of GC is still poor, partially on account of up to 50% of GC patients have unspecific gastrointestinal symptoms, and alarm symptoms are usually present at advanced stage in most cases^3^. Peritoneal metastases are common in advanced GC patients which usually leads to poor prognosis^4^. So far, there are still no effective treatments for peritoneal metastases due to little understandings on the underlying mechanisms.

A monolayer of peritoneal mesothelial cells (PMCs) that lines the peritoneal cavity has been reported to be able to undergo mesothelial-to-mesenchymal transition (MMT), an important morphological change in peritoneal metastases^5^. Emerging evidence shows that MMT of PMCs was observed in peritoneal dissemination and promoted early cancer metastasis^6–9^. Many studies have demonstrated that, through MMT, PMCs obtain enhanced invasive capacity and attach to cancer cells, and also acquire the capacity to synthesize inflammatory and angiogenic factors, such as fibroblast growth factor, vascular endothelial growth factor and growth factor, which have a growth promotion effect in cancer cells^10–12^. However, the molecular mechanisms that cause MMT of PMCs have yet to be fully explained.

Exosomes were first described as 5′-nucleotidase activity microvesicles by Trams et al. in 1981^13^ which are now identified by a few characteristics, such as, 30–150 nm in diameter, round or cup-shaped morphology, lipid composition and double lipid layer^14^. Exosomes contain proteins, lipids, miRNA, mRNA, and DNA, and enable the target cells to change gene expression^15^. Specifically, GC-derived exosomes have been proved to induce MMT of PMCs via MAPK/ERK pathway^16^. Furthermore, Tokuhisa M investigated exosomal miRNA profiles in peritoneal fluid and found that miR-21-5p had a high expression in serosal invasion GC. Their findings suggest that miR-21-5p may serve as biomarkers of peritoneal metastasis after GC resection^17^. Therefore we hypothesized that GC-derived exosomal miR-21-5p induces PMCs MMT, which leads to peritoneal metastasis.

In this study, our experimental results indicated that GC-derived exosomal miR-21-5p can convert PMCs into MMT via targeting SMAD7, leading to the increased invasion of PMCs and attachment to tumor cells. Finally, it promoted GC peritoneal metastasis. In addition, our results suggested that TGFβ/Smad pathway might be involved in this pathological process.

## Results

### Characterization of GC cells-derived exosomes and internalization

**Fig. 1:**
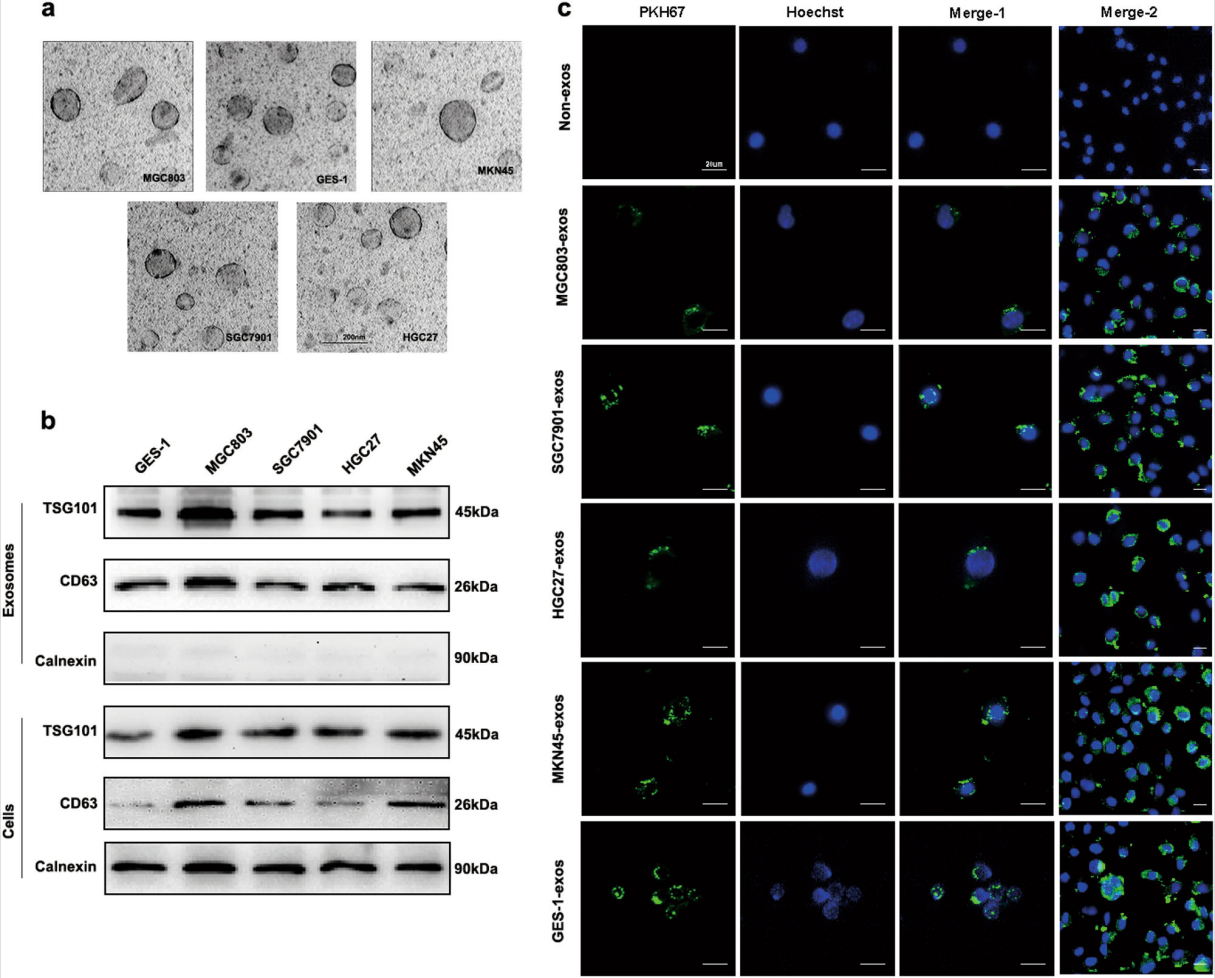
Characterization of GC cells-derived exosomes and internalization. **a** Exosomes purified from culture supernatant of the four GC cells and GES-1 cells were detected by TEM (Scale bar, 200 nm). **b** Exosomes marker proteins CD63 and TSG101 were identified by western blot. Calnexin was used as an internal reference. **c** Exosomes purified from culture supernatant of the four GC cells and GES-1 cells were labeled by PKH67, and HMrSV5 co-cultured with these exosomes or non-exosomes were observed under confocal microscopy (Scale bar = 20 µm). Merge-2 group was a number of pictures captured at lower magnification than merge-1. Non-exosomes group was used as the negative control. Each experiment was repeated at least three times

Exosomes from supernatant of four GC cell lines (MGC803, MKN45, HGC27, and SGC7901) and normal human gastric epithelial cell line GES-1 were isolated and evaluated by TEM and western blot. As shown in Fig. 1a, TEM showed that exosomes had the typical round or cup-shaped morphology, measuring about 100 nm in diameter. Furthermore, western blot profile showed the presence of known exosome markers, including proteins CD63 and TSG101^18^, in both exosomes and cells fractions. The protein “calnexin” was used as the negative control which was confirmed absent in exosomes but present in cells^19^ (Fig. 1b).

To identify whether GC-derived exosomes could be taken up by recipient cells, we used PKH67 dye (green) and hoechst 33342 dye (blue) to label exosomes and cell nucleus, respectively. HMrSV5 cells co-cultured with non-exosomes were used as the negative control. As shown in Fig. 1c, PKH67 labeled exosomes dispersed in the cytoplasm of HMrSV5 cells.

### GC-derived exosomes induce MMT of HMrSV5 cells in vivo and foster peritoneal metastasis

**Fig. 2:**
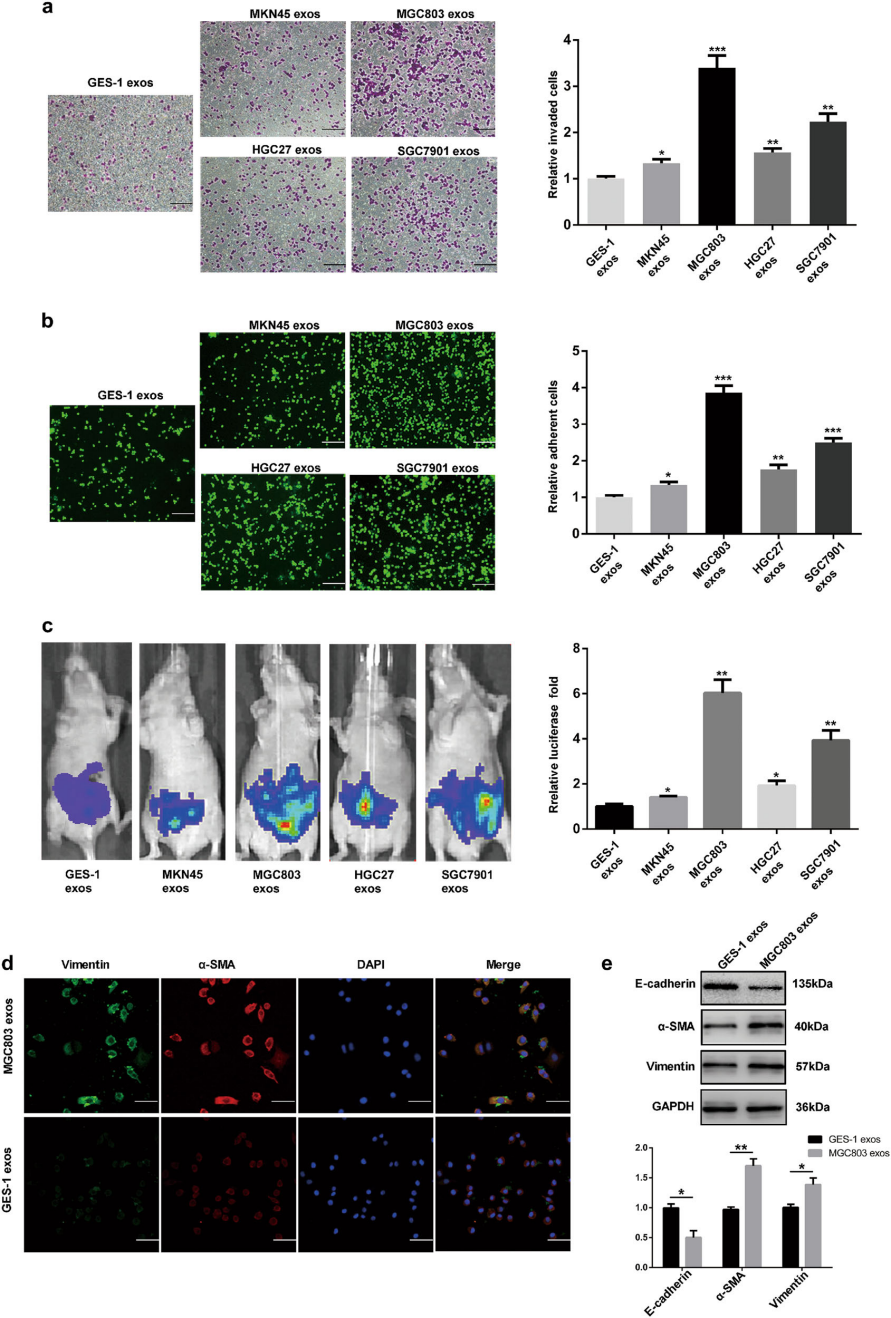
GC-derived exosomes induce MMT of HMrSV5 cells in vivo and promote peritoneal metastasis. **a** Invasion assays of HMrSV5 cells which were pretreated with 200 µg exosomes isolated from culture supernatant of four GC cells and GES-1 cells. Scale bar = 100 µm. **b** Adhesion assays of HMrSV5 cells which were pretreated with 200 µg exosomes. Scale bar = 100 µm. Calcein AM labeled BGC823 cells were added to investigate the adhesion ablitity of HMrSV5. **c** Bioluminescence images of tumor peritoneal dissemination. **d** MMT protein markers (Vimentin and α-SMA) were identified by immunofluorescence assays in exosomes-treated group and control group. Scale bar = 50 µm. **e** Western blot was performed to further confirm the MMT protein markers (including E-cadherin, α-SMA, and Vimentin). GES-1 cells-derived exosomes were used as the negative control in vivo and in vitro. GAPDH was used as an internal reference for western blot. Each experiment was repeated at least three times. All the data were expressed as mean ± SEM, and the results of multiple comparisons were corrected with Bonferroni method (Student’s *t*-test *P < 0.05, **P < 0.01, ***P < 0.001)

As we have speculated that exosome-induced MMT may play an important role in peritoneal metastasis, we firstly evaluated the different abilities to induce MMT among four GC cell line-derived and GES-1 cells-derived exosomes in vitro (GES-1 cells-derived exosomes were used as the negative control). For invasion assays, HMrSV5 cells were co-cultured with four GC cell line-derived exosomes respectively in treatment group and GES-1 cells-derived exosomes in control group, and then the invasion ability of HMrSV5 cells were evaluated by using matrigel-coated transwell inserts. As shown in Fig. 2a, more cells invaded in GC cell line group were compared with the control group. For adhesion assays, HMrSV5 cells were pretreated by using the same method mentioned above, and then BGC823 was used to test the attachment of HMsSV5 cells to cancer cells. Interestingly, in four GC cell lines groups, more BGC823 cells were adhered to a dense layer of HMrSV5 cells (Fig. 2b). Meanwhile, we assessed the effect of exosomes on peritoneal implantation in vivo by injecting exosomes from MKN45, MGC803, HGC27, SGC7901, and GES-1 into the peritoneal cavity of mice (GES-1 cells-derived exosomes were used as the negative control). As shown in Fig. 2c, GC cell lines-derived exosomes markedly contributed to the peritoneal dissemination. All the resluts of multiple comparisons were corrected with the Bonferroni method. Thus, MGC803 was selected for further experimental study based on the above findings in vivo and in vitro. In order to further validate the effect of GC-derived exosomes on MMT of HMrSV6, immumofluorescence and western blot were used to assess the protein expressions of MMT markers, including E-cadherin, α-SMA, Vimentin (Fig. 2d, e. GES-1 cells-derived exosomes were used as the negative control for immunofluorescence and GAPDH was used as an internal reference for western blot). Our findings suggested that MGC803-derived exosomes induce MMT of HMrSV5 cells and promote GC peritoneal metastasis.

### GC-derived exosomes transfer miR-21-5p from donor cells to recipient cells

**Fig. 3:**
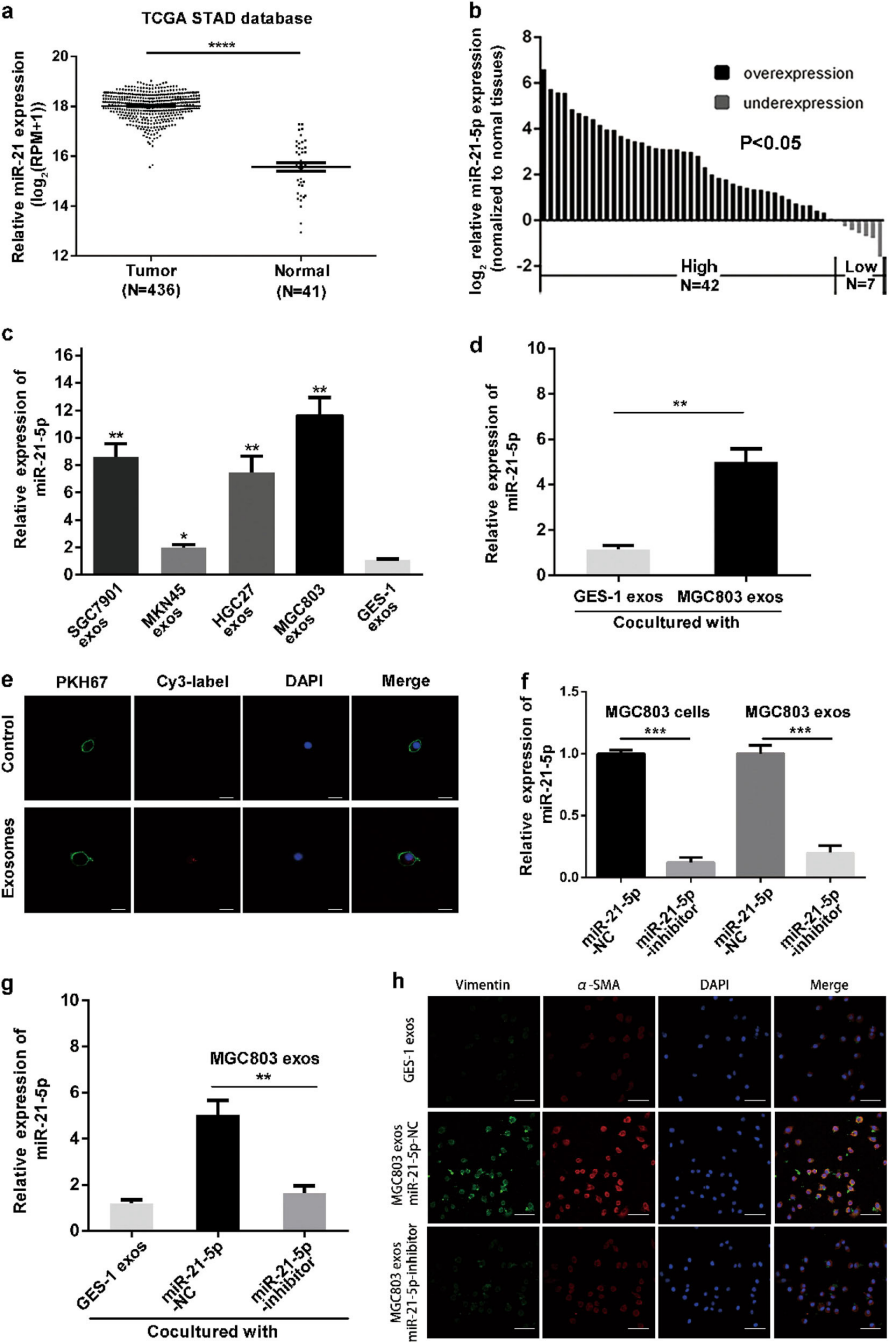
GC-derived exosomes transfer miR-21-5p from donor cells to recipient cells. **a** The expression levels of miR-21-5p in TCGA dataset between GC tissues and normal tissues. All normalized expression values were expressed in log_2_((RPM + 1)). **b** The expression levels of miR-21-5p in 49 paired GC tissues and adjacent normal tissues. All normalized expression values were expressed in log_2_2 ^−ΔΔCT^. **c** qRT-PCR detection of miR-21-5p expression in exosomes derived from four GC cell lines and GES-1 cells. GES-1 cells-derived exosomes were used as the negative control. **d** qRT-PCR detection of miR-21-5p expression in HMrSV5 co-cultured with MGC803-derived exosomes and GES-1 cells-derived exosomes (negative control). **e** Exosomes with Cy3-labeled miR-21-5p or without Cy3-labeled miR-21-5p were added to HMrSV5 cells. MiR-21-5p without Cy3-label was used as the negative control. The fluorescence signals were detected under confocal microscope. Scale bar = 20 µm. **f** The levels of miR-21-5p in HMrSV5 cells transfected with miR-21-5p-NC or miR-21-inhibitor and in the related exosomes. **g** qRT-PCR detection of miR-21-5p expression in HMrSV5 cells co-cultured with exosomes derived from GES-1 cells or MGC803 cells transfected with miR-21-5p-NC or miR-21-5p-inhibitor. **h** Immunofluorescence assays were performed to measure the MMT protein markers (Vimentin and α-SMA). Scale bar = 50 µm. MiR-21-5p-NC group was used as the negative control. Each experiment was repeated at least three times. All the data were expressed as mean ± SEM, and the results of multiple comparisons were corrected with Bonferroni method (Student’s *t*-test *P < 0.05, **P < 0.01, ***P < 0.001)

As we have found that GC-derived exosomes could induce MMT of HMrSV5, we next investigated how GC-derived exosomes activate MMT. Recent studies have indicated that there are abundant miRNAs encapsulated in exosomes, and these miRNAs play an important role in cell–cell communications^20^. In addition, exosomal miR-21-5p has been proved to be a crucial miRNA in cancer metastasis^21,22^, and is enriched in GC-derived exosomes^23^. Therefore, we hypothesized that GC-derived exosomal miR-21-5p mediate MMT activation. We firstly investigated the correlation between the miR-21-5p levels and GC. The TCGA-STAD/Xena_Matrices/TCGA-STAD.mirna.tsv showed that the mean expression value of miR-21-5p in GC tissues was 18.02 ± 0.03 compared to 15.58 ± 0.17 in normal tissues (Fig. 3a). Moreover, 49 paired GC patients’ tissues including tumor and adjacent normal tissues were used to further validate the expression profile (Fig. 3b).

Then we analyzed the correlation between miR-21-5P expression levels and the clinicopathological features of GC patients (age, gender, tumor size, histology grade, T grade, N grade, TNM stage). According to miR-21-5p median expression value, GC patients were divided into two groups: high-miR-21-5p group and low-miR-21-5p group. As shown in Table 1, mirR-21-5p level was significantly correlated with T grade (*P* = 0.028), N grade (*P* = 0.015) and TNM stage (*P* = 0.046). To preliminary investigate the correlation between miR-21-5p expression in exosomes and MMT, we used quantitative real-time polymerase chain reaction (qRT-PCR) to measure the levels of miR-21-5p in exosomes. After Bonferroni’s correction applied to our results, we found that the levels of miR-21-5p in exosomes derived from four GC cell lines were still significantly higher than that of GES-1 cells, which were consistent with the previous results in vivo and in vitro (Fig. 3c). To explore the effect of GC-derived exosomes on recipient cells’ miRNA expression, we used qRT-PCR to confirm the miR-21-5p levels of HMrSV5 cells, which was co-cultured with MGC803-derived exosomes and GES-1-derived exosomes. As shown in Fig. 3d, the intracellular miR-21-5p levels markably increased after co-culturing with MGC803-derived exosomes.

**Table1:** The correlation between miR-21-5p expression levels and the clinicopathological features of GC patients

Characteristics	Number	miR-21-5p expression		*P*-value
		High group	Low group	
Age (years)				
<60	17	11	6	0.163
≥60	32	14	18	
Gender				
Male	37	20	17	0.456
Female	12	5	7	
Size (cm)				
<3	19	7	12	0.114
≥3	30	18	12	
Histology grade				
Well + moderate	15	5	10	0.100
Poor + undifferentiated	34	20	14	
T grade				
T1 + T2	24	8	16	0.015^*^
T3 + T4	25	17	8	
N grade				
Present (N1 + N3)	27	18	9	0.015^*^
Absent (N0)	22	7	15	
TNM stage				
I + II	32	13	19	046^*^
III + IV	17	12	5	

^*^P < 0.05, **P < 0.01, ***P < 0.001

To further investigate whether the MGC803-derived exosomes could transfer miR-21-5p from MGC803 cells to HMrSV5 cells, we transiently transfected MGC803 with either Cy3-labeled miR-21-5p (red) or negative control. HMrSV5 cells were pretreated with PKH67 (green) and Hoechst 33342 (blue) respectively, and then the exosomes from MGC-803 culture supernatant were isolated and added to HMrSV5 cells culture supernatant. As shown in Fig. 3e, red fluorescent signals could be observed in HMrSV5 cells in Cy3-labeled group but not in negative control group.

For further exploring whether the increase of miR-21-5p levels in HMrSV5 cells was directly due to uptaking of the exosomal miR-21-5p derived from MGC803 cells. We constructed two stable cell lines by transfecting MGC803 cells with lentvirus contructs (miR-21-5p inhibitors or negative control for inhibitors). To verify the efficiency of transfection, the expression of miR-21-5p in MGC803 cells and exosomes was meatured by qRT-PCR. As shown in Fig. 3f, the levels of miR-21-5p in cells and exosomes were significantly downregulated in miR-21-5p-inhibitor group compared with miR-21-5p-NC group. Then, HMrSV5 cells were co-cultured with exosomes derived from MGC803 miR-21-5p-NC or miR-21-5p-inhibitor. As shown in Fig. 3g, the expression of miR-21-5p in miR-21-5p-inhibitor group was distinctly decreased relative to miR-21-5p-NC group. Similarly, the result of immunofluorescence was consisitent with above findings (Fig. 3h). Altogether, these above results demonstrated that exosomal miR-21-5p could be transferred from MGC803 cells to HMrSV5 cells, and eventually lead to siginificantly increase of miR-21-5p levels.

### MiR-21-5p induces MMT in HMrSV5 cells in vitro

**Fig. 4:**
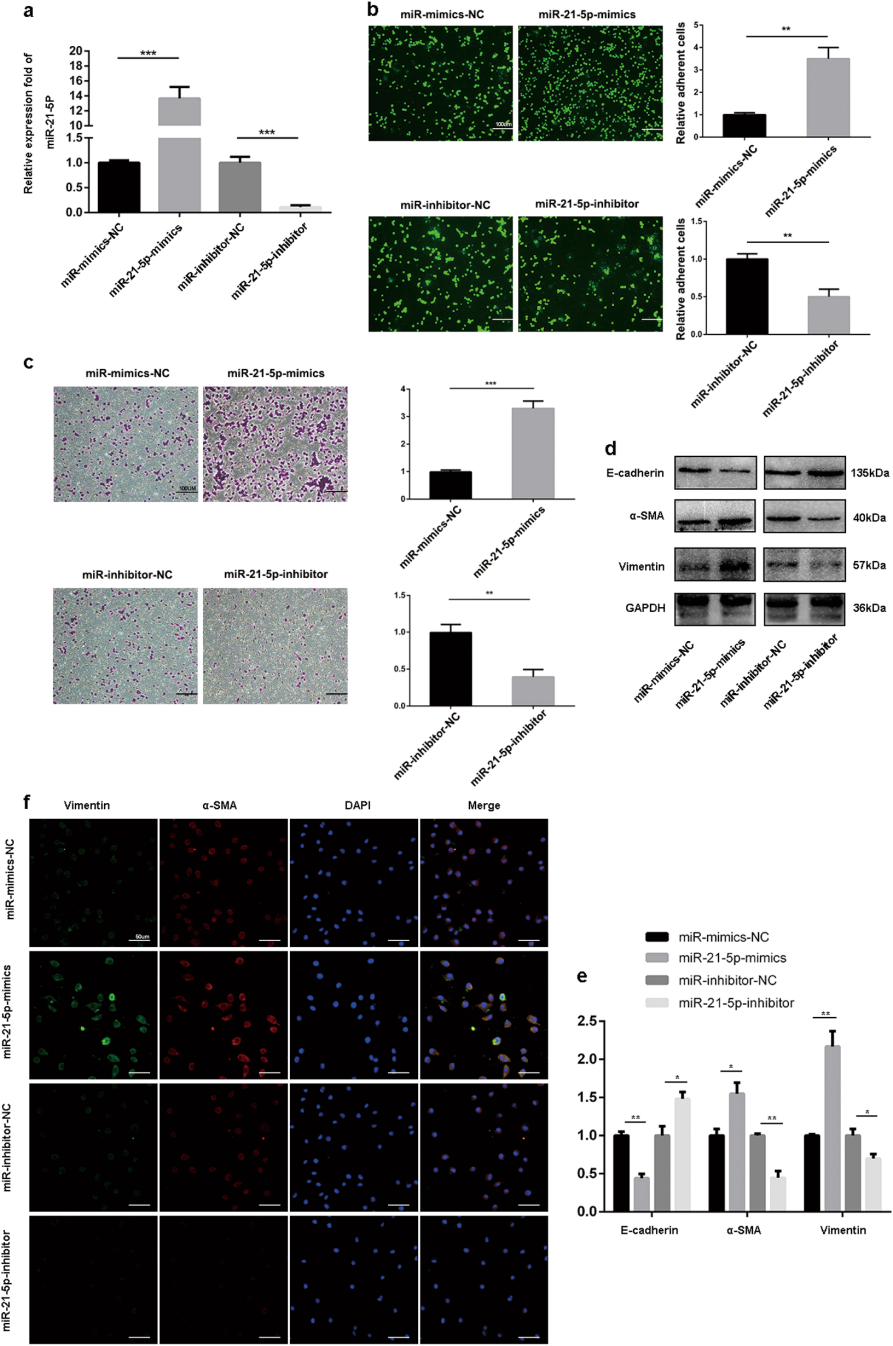
MiR-21-5p induces MMT in HMrSV5 cells in vitro. **a** qRT-PCR detection of miR-21-5p expression in HMrSV5 transfected with miR-21-5p-mimics, mimics-NC, miR-21-5p-inhibitor and inhibitor-NC. Mimics-NC was the negative control for miR-21-5p-mimics and inhibitor-NC was the negative control for miR-21-5p-inhibitor. **b, c** Adhesion (Scale bar = 100 µm) and invasion (Scale bar = 100 µm) assays showed the effect of miR-21-5p on the HMrSV5 in vitro. **d, e** Western blot assays were performed to confirm the MMT of HMrSV5. GAPDH was used as an internal control. **f** Immunofluorescence assays were performed to confirm the MMT of HMrSV5. Scale bar = 50 µm. Each experiment was repeated at least three times. All the data were expressed as the mean ± SEM (Student’s *t*-test *P < 0.05, **P < 0.01, ***P < 0.001)

To further investigate whether miR-21-5p could mediate MMT of HMrSV5 cells, HMrSV5 cells were transfected diferent lentvirus contructs, including miR-21-5p mimics, negative control for mimics, miR-21-5p inhibitors or negative control for inhibitors. As shown in Fig. 4a, miR-21-5p levels in miR-21-5p-mimics group was significantly increased than miR-mimics-NC group, and miR-21-5p-inhibitor group showed markedly lower miR-21-5p levels than miR-inhibitor-NC. Next, adhesion and invasion assays were performed to evaluate the abilities of invasion and attachment to tumor cells of HMrSV5 cells transfected with mimics and inhibitor lentivirus constructs. As shown in Fig. 4b, more cells were adhered to HMrSV5 cells in mimics group compared to negative control group, and miR-21-5p inhibitor significantly decreased the numbers of the adhered cells. For invasion assays, overexpression of miR-21-5p markedly increased invasion of HMrSV5; in contrast, HMrSV5 cells transfected with miR-21-5p-inhibitor showed a marked decrease in invasion cells compared to inhibitor-NC group (Fig. 4c).

Finally, we used immunofluorescence (IF) and western blot to detect the changes of expression of MMT protein markers (including E-cadherin, α-SMA, and Vimentin). Western blot assays revealed that miR-21-5p-mimics group exhibited significantly higher α-SMA and Vimentin levels and lower E-cadherin levels compared with mimics-NC group. In contrast, compared to inhibitor-NC group, miR-21-5p-inhibitor group showed higher E-cadherin levels and lower α-SMA and Vimentin levels. Moreover, immunofluorescence assays further verified the influence of miR-21-5p in the changes of MMT protein markers (Fig. 4e, f). In summary, these results indicated that miR-21-5p could induce MMT in HMrSV5 cells in vivo.

### MiR-21-5p directly targets SMAD7-3′-UTR in HMrSV5 cells

**Fig. 5:**
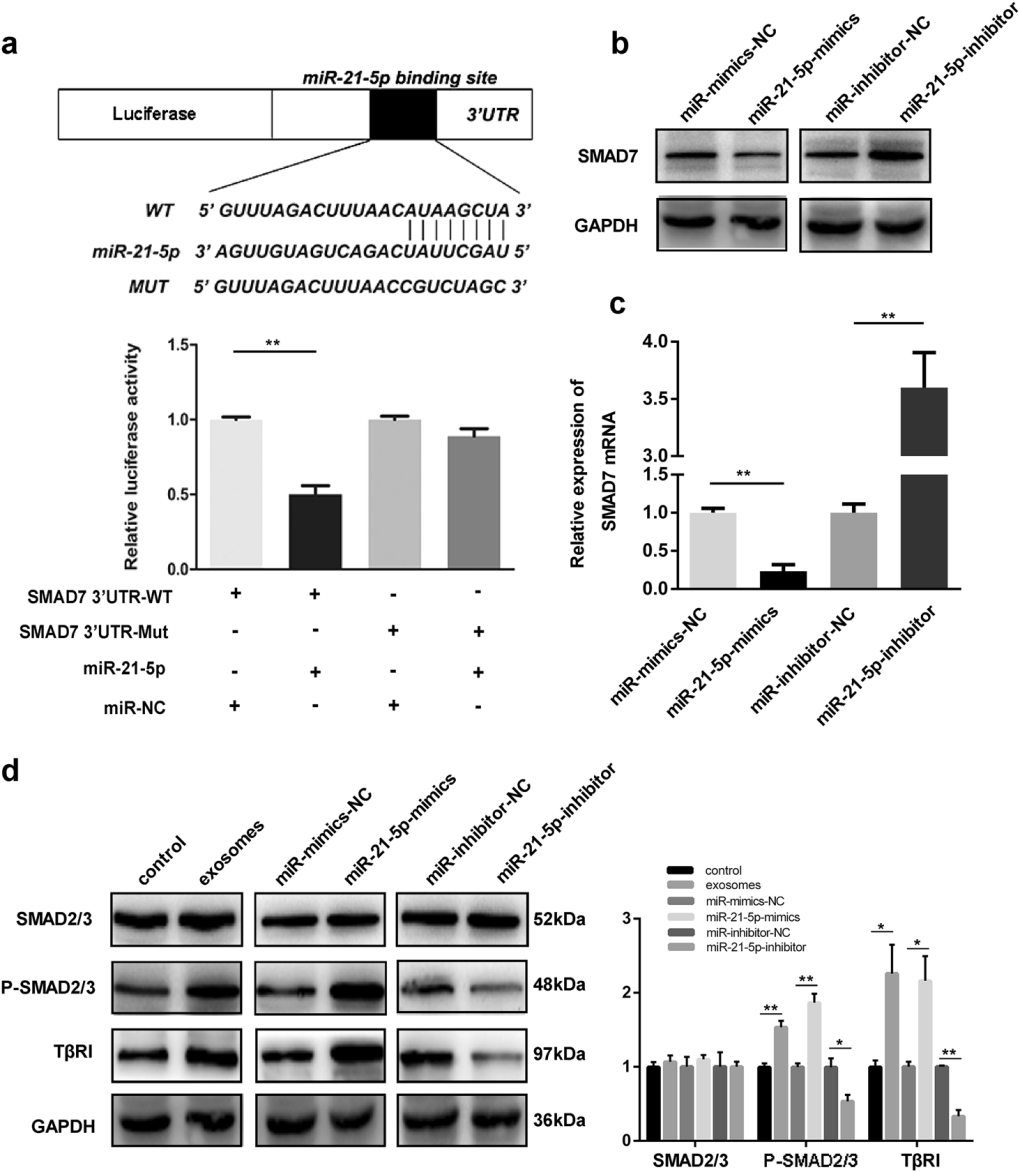
MiR-21-5p directly targets SMAD7-3′-UTR in HMrSV5 cells. **a** Luciferase reporter assay results indicated that miR-21-5p directly bind to SMAD7 3’UTR. **b** Western blot was performed to measure the protein levels of SMAD7 in different groups. **c** qRT-PCR detection of SMAD7 mRNA in HMrSV5 cells transfected with miR-21-5p-mimics, mimics-NC, miR-21-5p-inhibitor and inhibitor-NC. **d** Western blot analysis for the protein levels of SMAD2/3, p-SMAD2/3, and TβRI in different groups. GAPDH was used as an internal reference. Each experiment was repeated at least three times. All the data were expressed as mean ± SEM (Student’s *t*-test *P < 0.05, **P < 0.01, ***P < 0.001)

To explore how miR-21-5p exerts its function in MMT, we used TargetScan (http://www.targetscan.org/), miRanda (http://www.microrna.org/microrna/home.do) and PicTar (http://pictar.mdc-berlin.de/) to predict target genes of miR-21-5p. Then, SMAD7 was identified as a potential target of miR-21-5p, which has been proved to play an important role in EMT process^24^. Then, dual luciferase reporter assays were performed to validate further whether SMAD7 was a direct target of miR-21-5p. Mutated-type (MUT) and wild-type (WT) SMAD7 3′-UTR sequences (the former containing site-directed mutations in the putative miR-21-5p target sites) were cloned into the luciferase vectors. As shown in Fig. 5a, wild-type binding site vector markedly decreased the luciferase activity in the presence of miR-21-5p compared to control group. However, mutated-type binding site vector did not result in significantly decreased luciferase activity in the presence of miR-21-5p. These data revealed that SMAD7 is a direct target of miR-21-5p in HMrSV5.

Next, we used qRT-PCR and western blot to investigate how miR-21-5p influences SMAD7. Western blot assays revealed that miR-21-5p-mimics group exhibited decreased SMAD7 protein level and miR-21-5p-inhibitor group showed increased SMAD7 protein level compared with negative control group (Fig. 5b). We then measured SMAD7 mRNA levels by qRT-PCR assays, and we found that there were significant changes of SMAD7 mRNA levels in miR-21-5p-mimics group and miR-21-5p-inhibitor group (Fig. 5c). These results suggested that miR-21-5p suppresses SMAD7 protein expression by degrading the corresponding mRNA.

Emerging evidence suggested that TGF-β/Smad pathway plays an important role in EMT and MMT^9,25^. In addition, SMAD7 has been proved to suppress TGF-β/Smad activity by binding SMAD2/3 and prevent their activation upon TGF-β1 stimulation in cancer-associated fibroblasts (CAFs) formation^26^. Thus, to investigate whether miR-21-5p mediates TGF-β/Smad pathway by targeting SMAD7, western blot assays were used to measure the SMAD2/3, phosphorylated-SMAD2/3 (P-SMAD2/3) and TGF-β type I receptor (TβRI) protein levels. As shown in Fig. 5d, P-SMAD2/3 and TβRI protein level exhibited significant increases in exosomes groups and miR-21-5p-mimics group compared to negative control group; but in miR-21-5p-inhibitor group, P-SMAD2/3 and TβRI protein level was lower than inhibitor-NC group. All these data suggested that miR-21-5p might mediate TGF-β/Smad pathway via targeting SMAD7.

### MiR-21-5p promotes MMT of HMrSV5 cells via targeting SMAD7

**Fig. 6:**
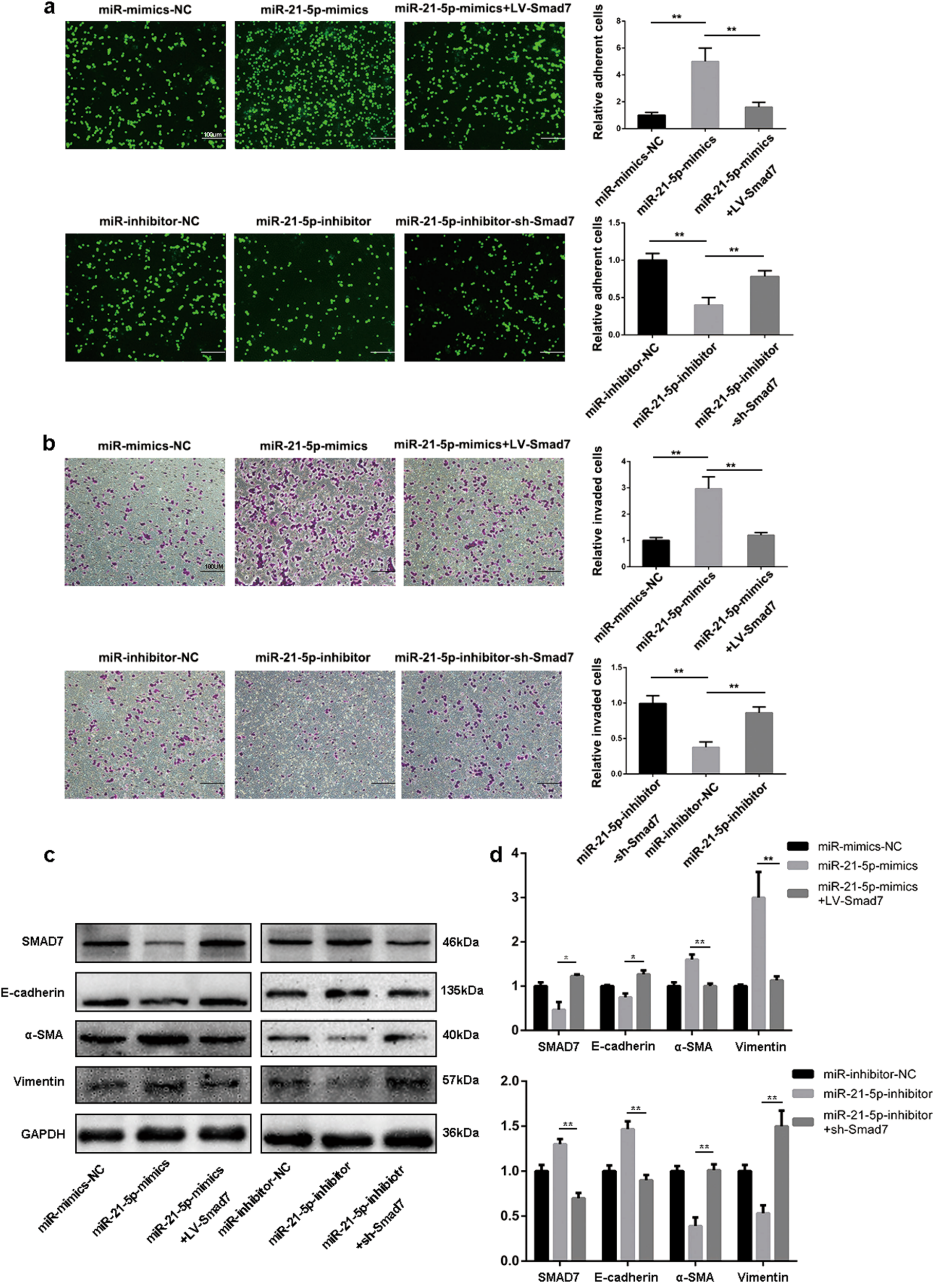
MiR-21-5p promotes MMT of HMrSV5 via targeting SMAD7. **a**, **b** To investigate the rescue function of SMAD7, adhesion (Scale bar = 100 µm) and invasion (Scale bar = 100 µm) assays were performed in HMrSV5-miR-21-5p-mimics or HMrSV5-miR-21-5p-inhibitor cells which were transfected with LV-SMAD7 or sh-SMAD7 without its 3’UTR. The negative controls for miR-21-5p-mimics + LV-SMAD7 and miR-21-5p-inhibitor + sh-SMAD7 were miR-21-5p-mimics and miR-21-5p-inhibitor, respectively. **c**, **d** The alteration of protein levels (including SMAD7, E-cadherin, α-SMA and Vimentin) were meatured by western blot. GAPDH was used as a loading control. Each experiment was repeated at least three times. All the data were expressed as the mean ± SEM, and the results of multiple comparisons were corrected with Bonferroni method (Student’s *t*-test *P < 0.05, **P < 0.01, ***P < 0.001)

Results above revealed that SMAD7 was a direct target of miR-21-5p, but whether miR-21-5p induced MMT via mediating SMAD7 expression is still not clear. Thus, we used lentiviral vectors LV-SMAD7 to transfect HMrSV5 cells which have been transfected with miR-21-5p-mimics vector and miR-21-5p-inhibitor HMrSV5 cells were transfected with lentiviral vectors sh-SMAD7. The negative controls for miR-21-5p-mimics + LV-SMAD7 and miR-21-5p-inhibitor + sh-SMAD7 were miR-21-5p-mimics and miR-21-5p-inhibitor, respectively. After Bonferroni correction, adhesion assays and invasion assays revealed that LV-SMAD7 effectively counteracted the increase of adhesion and invasion abilities resulting from miR-21-5p overexpression in HMrSV5 cells. Likewise, sh-SMAD7 reversed the decrease of adhesion and invasion abilities resulting from miR-21-5p knockdown (Fig. 6a, b). Furthermore, western blot assays results indicated that SMAD7 and E-cadherin protein levels were reversed in miR-21-5p-mimics-LV-SMAD7 group compared to miR-21-5p-mimics. Similarly, α-SMA and Vimentin protein levels were counteracted in miR-21-5p-inhibitor-sh-SMAD7 (Fig. 6c, d). Collectively, ourfindings confirmed the hypothesis that miR-21-5p promotes MMT of HMrSV5 by targeting SMAD7.

### MiR-21-5p promotes GC peritoneal metastasis by inducing MMT in vivo

**Fig. 7:**
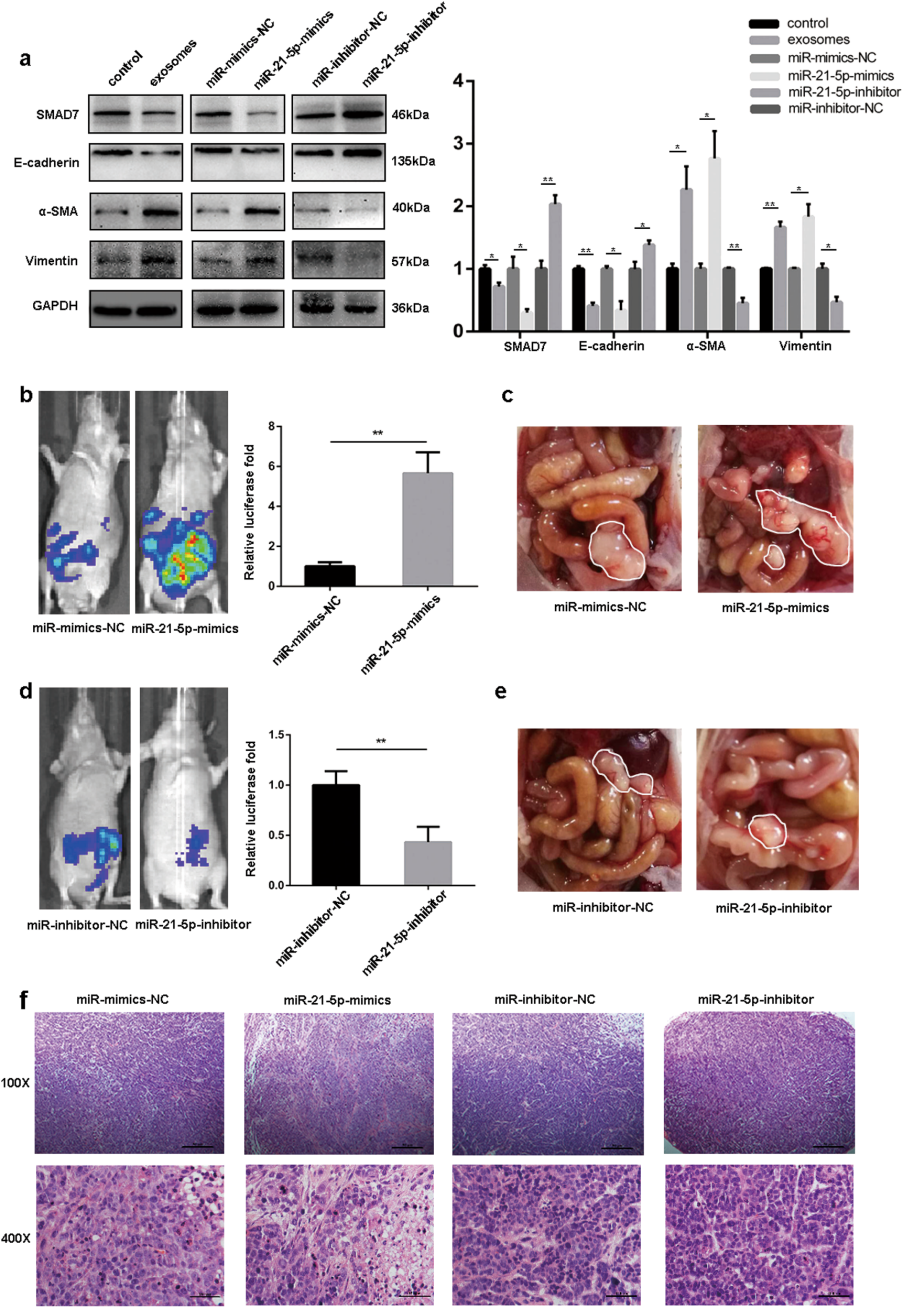
MiR-21-5p promotes GC peritoneal metastasis by inducing MMT in vivo. **a** Western blot was performed to analyse the expression levels of SMAD7, E-cadherin, α-SMA, and Vimentin in peritoneal tissues from different groups. GAPDH was used as a loading control. **b**, **d** Bioluminescence images of tumor peritoneal dissemination in different groups, and the luciferase activity was analyzed. **c**, **e** Representative images were captured from different groups, and tumors were outlined in white. **f** HE stain for tumor tissues from each groups (Scale bar = 50 µm, 100X magnification; Scale bar = 100 µm, 400X magnification). Each experiment was repeated at least three times. All the data were expressed as mean ± SEM (Student’s *t*-test *P < 0.05, **P < 0.01, ***P < 0.001)

To further explore the potential influences of exosomal miR-21-5p in vivo, we generated a mouse model of peritoneal dissemination via peritoneum injection of GC-derived exosomes, NC-mimics lentivirus, miR-21-5p mimics lentivirus, NC-inhibitor lentivirus and miR-21-5p inhibitor lentivirus. To assess the microenvironment changes of peritoneum, peritoneal tissues of mice were collected for western blot assays 4 days after the inoculation. As shown in Fig. 7a, western blot analysis revealed that peritoneum pre-conditioned with GC-derived exosomes, miR-21-5p mimics lentivirus decreased E-cadherin level, increased α-SMA and Vimentin protein levels in the mesothelial monolayer, which indicated that MMT had taken place in the peritoneum. Additionally, the SMAD7 expression indicated that exosomal miR-21-5p induces MMT in peritoneum by suppressing SMAD7 protein level.

Next, to assess the peritoneal metastasis in mice, BGC823-luc-D3 cells were injected into the peritoneal cavity. 2 weeks after the inoculation, we found that luciferase activity of miR-21-5p-mimics group was significantly higher than that of mimics-NC group, while the opposite pattern of luciferase activity could be observed in miR-21-5p-inhibitor group (Fig. 7b, d). In addition, representative images of peritoneal metastasis were chosen to further verify tumor progression in the peritoneum (Fig. 7c, e). Finally, tumor tissues from each group were confirmed by HE staining (Fig. 7f).

## Discussion

Peritoneal metastases, the most common way of dissemination in GC, have always been highly concerned by researchers. In 1889, Stephen Paget proposed “seed and soil” theory in which he stated that how the metastasis occurs not only depends on tumor cell itself (seed), but also requires a receptive environments (soil)^27^. More recently, MMT of PMCs was found in various types of tumors with peritoneal metastasis. PMCs undergoing MMT would cause structural alteration of peritoneum by promoting matrix remodeling and angiogenesis, and then favor invasion, growth and adhesion of the tumor cells by metastasis^6,28,29^. Thus, MMT might be a therapeutic target to prevent peritoneal metastasis.

In the last few years, accumulating evidence revealed that exosomes could be involved in various pathological processes including resistance to tumor treatment and metastasis by intercellular communication^30,31^. MicroRNAs (miRNAs) are small non-coding RNAs with 20–24 nucleotides which bind within the 3′-UTR of mRNAs, and then reduce these RNAs’ encoded proteins^32^. Previous studies have revealed that several miRNAs were involved in cancer peritoneal metastasis (including miR-516a-3p, miR-409, miR-200a/b, and miR-193b)^22,33–35^. Furthermore, emerging evidence indicates that some specific miRNAs could be transferred to recipient cells to decrease the expression of target genes. In liver cancer, tumor-derived exosomes transfer miR-1247-3p from donor cells to recipient cells and induces CAFs activation by targeting B4GALT3, finally leading to lung dissemination^36^. For gastric cancer, recent studies have demonstrated that tumor-derived exosomes promote peritoneal metastasis by inducing MMT of MCs^16,37^; however, these studies did not investigate which proteins, miRNA, or DNA were transferred by exosomes to recipient cells and induced MMT process.

In this study, we firstly identified the purified exosomes from four GC cell lines and GES-1 cells by TEM, uptaken by HMrSV5 cells and exosomes marker protein by western blot assays. Then, purified exosomes were used in adhesion, invasion assays, and tumor peritoneal dissemination experiments. These results showed that GC-derived exosomes could increase the abilities of invasion and adhesion in HMrSV5 and lead to peritoneal metastasis in vivo. Additionally, MGC803-derived exosomes showed the strongest promotion ability in promoting MMT of HMrSV5 in vitro and peritoneal dissemination in vivo. Thus, MGC803-derived exosomes were selected for further studies.

Previous studies have illuminated that miR-21-5p was high levels and be able to promote tumor invasion, intravasation and metastasis in various types of cancers (including colorectal cancer, GC, breast cancer and brain cancer), and exosomal miR-21-5p was also identified to be involved in oral squamous cell carcinoma metastasis^22,35,38^. Furthermore, Tokuhisa M has found that exosomal miR-21-5p expression in peritoneal lavage fluid samples from invasive GC was higher than that of non-invasive GC, which suggested that exosomal miR-21-5p might be involved in GC peritoneal metastasis. Thus, further exploration on the role of exosomal miR-21-5p in GC peritoneal metastasis is urgently needed. In our study, TCGA miRNA expression data indicated that miR-21-5p expression was markedly higher in tumor goup than that in normal controls. Similarly, 49 paired GC tissues showed that miR-21-5p expression was significantly higher in GC tissues than that in adjacent normal tissues. Also, the result of qRT-PCR in exosomes derived from GC cell lines and GES-1 cells suggested that miR-21-5p in exosomes may be the key factor for promoting peritoneal metastasis. Furthermore, we confirmed that miR-21-5p was indeed transferred to HMrSV5 cells, resulting in an increased expression of miR-21-5p in HMrSV5 cells. Our results also suggested that overexpression of miR-21-5p could induce MMT of HMrSV5 in vitro and promoted peritoneal metastasis in vivo, and the opposite results could be found when the miR-21-5p expression was knocked down.

To further explore the mechanisms of miR-21-5p induced MMT in HMrSV5, we used bioinformatics analysis to predict miR-21-5p’s putative targets. Then, SMAD7 was chosen for further study from the candidate target genes as previous studies have demonstrated that SMAD7 was associated with tumor invasion and metastasis^33,39^. In our study, we used luciferase reporter assays to confirm that miR-21-5p directly bound to SMAD7 within 3′-UTR and led to degradation of SMAD7 mRNAs. Additionally, results of invasion, adhesion assays and western blot assays revealed that miR-21-5p induced MMT of HMrSV5 via targeting SMAD7.

According to previous studies, TGF-β pathway might promote MMT in peritoneal metastasis, and blocking TGF-β pathway could reduce peritoneal dissemination^6^. SMAD2/3 are important proteins in TGF-β pathway, which are activated by TβRI, and then transform into phosphorylated forms. Then, phosphorylated SMAD2/3 regulate the expression of target genes in the nucleus^40^. Furthermore, studies have suggested that knockdown of SMAD3 expression could reduce the growth and metastasis of intraperitoneal tumor in mice via blocking TGF-β/Smad pathway in MMT. Thus, TGF-β/Smad pathway inhibitors may play an important role in reducing peritoneal metastasis by blocking MMT via preventing SMAD2/3 from being phosphorylated. Previous studies have indicated that SMAD7 mitigates TGF-β/Smad pathway via recruiting E3-ubiquitin ligase SMURF2 to TβRI for degradation^41^. Our studies also revealed that exosomal miR-21-5p activated TGF-β/Smad pathway by mitigating the inhibitory action of SMAD7.

In conclusion, our study demonstrates that GC-derived exosomes could promote peritoneal metastasis by inducing MMT. The data also indicated that exosomal miR-21-5p transferred from GC cells could induce MMT in MCs via targeting SMAD7 and reduce tumor peritoneal metastasis. Based on all of the above evidences, exosomal miR-21-5p may be a novel therapeutic target for GC peritoneal metastasis.

## Methods

### Patients and tissue samples

Forty-nine patients diagnosed with GC who underwent radical gastrectomy were enrolled in this study at the Department of General Surgery, First Affiliated Hospital, Nanjing Medical University, China from January 2016 to December 2017. Informed consent was obtained from each patient or relatives, and the protocol was approved by the Ethical Committee of Nanjing Medical University.

### Cell lines and cell culture

The human GC cell lines MGC803, BGC823, MKN45, HGC27, and SGC7901 and the normal human gastric epithelial cell line GES-1 were purchased from the Cell Center of Shanghai Institutes for Biological Sciences (Shanghai, China). The human peritoneal mesothelial cell line HMrSV5 was kindly provided by Professor Pierre Ronco, Hospital Tenon (Paris, France) who originally established this cell line by using retrovirus to transfect primary human PMCs with SV40 large-T antigen^42^. HMrSV5 cell line has been applied to a lot of researches on peritoneum^43–45^. All cell lines were cultured in RPMI-1640 medium supplemented with 10% fetal bovine serum (FBS; WISENT, Canada) and antibiotics (1% penicillin/streptomycin, Gibco, USA) in a humidified atmosphere of 95% air and 5% CO_2_ at 37 °C.

### Exosome isolation and labeling

GC cells were cultured in RPMI-1640 supplemented with 10% exosome-free FBS for 48 h. The conditioned medium was collected and exosomes were isolated by using ExoQuick-TC^TM^ (System Biosciences, USA) according to the manufacturer’s instructions. The final exosome pellets were resuspended in PBS and stored at −80 °C for further experimental needs. According to the published literatures, the concentration of exosomes was determined by using bicinchoninic acid assay (BCA, Thermo Scientific, USA). Purified exosomes were labeled with the PKH67 green fluorescent linker Mini Kit (Sigma, USA) according to the manufacturer’s instructions.

### Transmission electron microscopy (TEM)

For TEM observation, a moderate amount of exosome suspension was placed on carbon-coated 300 mesh copper grids (Agar Scientific Ltd., Stansted, UK) for 2 min. The samples were fixed in 3% paraformaldehyde at 4 °C overnight and 1% osmium tetroxide at pH 7.2 for 1 h at room temperature (RT). After washing in double-distilled water for three times, samples were stained with uranyl acetate for 10 min and lead citrate for 5 min at RT. After air-drying, exosomes can be viewed with a FEI Tecnai T20 TEM, operated at 120 kV.

### Quantitative real-time polymerase chain reaction (qRT-PCR)

Total RNA was extracted from tissues, cultured cells and exosomes using the TRIzol reagent (Invitrogen) according to the manufacturer’s instructions. Then RNA was reverse transcribed into cDNA using PrimeScript RT Reagent (TaKaRa, Japan). PCR reactions were performed using a 7500 Realtime PCR System (Applied Biosystems, Carlsbad, CA, USA). All of the PCR reactions were run in triplicate. U6 small nuclear RNA (snRNA) was used as an internal control of miRNA levels. Expression of miR-21-5p was normalized to snRNA U6 and relative expression was calculated with the equation 2^–ΔΔCT^.

Primers of hsa-mir-21-5p, U6, SMAD7 and actin were as follows:

5′- TAGCTTATCAGACTGATGTTGA - 3′ (hsa-mir-21-5p, forward);

5′- CTCGCTTCGGCAGCACA - 3′ (U6, forward);

5′- AACGCTTCACGAATTTGCGT - 3′ (U6, reverse);

5′- GCTCCCATCCTGTGTGTTAA - 3′ (SMAD7, forward);

5′- TAGGTGTCAGCCTAGGATGGT - 3′ (SMAD7, reverse);

5′- GCATCGTCACCAACTGGGAC - 3′ (β-actin, forward);

5′- ACCTGGCCGTCAGGCAGCTC - 3′ (β-actin, reverse).

### Western blot analysis

The proteins extracted from cells, tissues and exosomes were separated by sodium dodecyl sulfate polyacrylamide gel electrophoresis (SDS-PAGE) and transferred to polyvinylidene fluoride (PVDF) membranes (Millipore). Membranes were post blocked with 5% fat-free dried milk in Tris-buffered saline for 2 h at RT and incubated at 4 °C overnight with anti CD63 (1:1,000, Abcam, Catalog # ab134045), anti TSG101 (1:1,000, Abcam, Catalog # ab125011), anti Calnexin (1:2,000, Abcam, Catalog # ab75801), anti α-SMA (0.1 µg/mL, R&D SYSTEMS Catalog # MAB1420), anti E-Cadherin (1:1,000, Abcam, Catalog # ab1416), anti Vimentin (1:1,000, Abcam, Catalog # ab8978), anti SMAD7 (1:1,000, Santa Cruz Biotechnology, Catalog # sc-365846), anti SMAD2/3 (1:2,000, Abcam, Catalog # ab202445), anti-phosphorylated SMAD2/3 (1:1000, Abcam, Catalog # ab63399), anti TβRI (1:1,000, Abcam, Catalog # ab220084) and GAPDH (1:200, Santa Cruz Biotechnology, Catalog # sc-47724) antibodies. After washing with TBST buffer three times, membranes were post incubated with HRP-conjugated anti-mouse or anti-rabbit IgG (diluted 1:2000 in TBST) at RT for 2 h, and then washed with TBST buffer three times again. Bands were scanned using enhanced chemiluminescence (ECL) detection system, and quantification was performed using Image J software. GAPDH was used as an internal control except for assays for verifying exosomes marker proteins.

### Immunofluorescence (IF) assay

HMrSV5 cells were cultured on collagen-coated glass coverslips (50,000 cells per well), and post fixed in 4% formaldehyde for 20 min at RT after washing with PBS at the time of harvest. After rewashing with PBS, the cells were permeabilized with 0.1% Triton X-100 for 10 min. 1% BSA in PBS was used to incubate with cells for blocking non-specific binding for 30 min. Then cells were treated with anti-N Cadherin (1:200, Abcam) and anti-Vimentin (1:100, Abcam) antibodies at 4 °C overnight. Subsequently, cells were incubated with the secondary Cy^TM^3-goat anti-rabbit IgG (Jackson, 1:100) and FITC-goat anti-mouse IgG (Jackson, 1:100) antibodies for 2 h, and post stained with DAPI for 15 min. A Nikon confocal microscope was used to take the fluorescence images of cells.

### HE staining

All tissues were fixed in 4% formalin in time. For HE staining, sections were firstly incubated with hematoxylin solution for 5 min after deparaffinization and rehydration, and then stained with five dips in 1% acid ethanol (1% HCl in 70% ethanol). Next, the sections were stained with eosin solution for 3 min, and then graded alcohol was used to dehydrate the sections followed by clearing in xylene. Representative images were captured using a fluorescence microscope.

### Luciferase reporter assay

HMrSV5 cells were co-transfected with 0.12 µg of pGL3-SMAD7 3′-UTR reporter plasmid containing the wild or mutated type miR-21-5p binding sequence (GeneScript, Nanjing, China) and together with 40 nM of miR-21-5p mimics or negative control oligoribonucleotides using Lipofectamine2000 (Invitrogen, USA) following the manufacturer’s instructions. For a reference control, 0.0 µg of Renilla luciferase expression plasmid was used to transfect the cells. After 36 h, dual luciferase reporter assays (Promega, E1910, WI, USA) were used to detect Firefly and Renilla luciferase activities, and then ratio of Firefly and Renilla luciferase activities can be used to calculate relative luciferase activity.

### Vector constructs, lentivirus production and cell transfections

LV2-hsa-miR-21-5p-mimics vector (miR-21-5p-mimics) and the LV2-hsa-miR-21-5p-inhibitor vector (miR-21-5p-inhibitor) constructed by lentiviral vectors (GenePharma, Shanghai, China). The negative control was LV2 empty lentiviral construct. Approximate 2 × 10^5^ cells were infected with 1 × 10^6^ lentivirus transducing units in the presence of 1 µg/ml polybrene. For getting stable transfected cell lines, transfected cells were selected by using cultures containing 5 µg/ml puromycin (Sigma, Aldrich) for 5 days. The LV-SMAD7, LV-SMAD7-shRNA which all containing the puromycin resistance sequence were constructed by GenePhama (Shanghai, China), and cells were transfected by the ways described above. The miR-21-5p expression was analyzed by Hairpin-it^TM^ miRNA qPCR Quantitation Kit, and SMAD7 expression was analyzed by qRT-PCR and western blot.

### Cell invasion assay

The invasion ability of cell was tested by using Matrigel-coated transwell inserts (BD Biosciences, Franklin Lakes, NJ, USA) according to the manufacturer’s instructions. All images were taken by a photo-microscope in three randomly selected fields, and cell invasion was quantified by blind counting.

### Tumor cell adhesion assay

According to previous studies, the ability of mesothelial cells’ attachment to tumor cells can be determined by adhesion assay^46^. HMrSV5 cells were seed in 96-well plates overnight to create a confluent monolayer. About 5 × 10^4^ per well BGC823 cells stained with 15 µM of calcein AM for 30 min according to the manufacturer’s instructions were added to 96-well and incubated for 3 h at 37 °C. Non-adherent tumor cells were washed by PBS, and then adherent cells were observed with a fluorescence microscope. All images were captured in three randomly selected fields, and cell adhesion was quantified by blind counting.

### Detection of Cy3-labeled miR-21-5p transfer

For directly observing the transfer of miRNA by exosomes, cancer cells were transfected with Cy3-labeled miR-21-5p or miR-21-5p without Cy3-labeled and miR-21-5p without Cy3-labeled was used as the negative control. Then, exosomes purified from them were incubated with HMrSV5 cells. After being washed with PBS, HMrSV5 cells were stained with PKH67 and Hoechest33342 at room temperature. Images were captured with a confocal microscope.

The Cy3-labeled hsa-miR-21-5p sequence used was:

5′-UCAACAUCAGUCUGAUAAGCUA-3′.

### In vivo tumor peritoneal dissemination model

For changing the microenvironment of the peritoneum, twenty BALB/c 4–6-week-old female nude mice were purchased from the Animal Center of NJMU and randomly allocated to five groups. Exosomes purified from cancer cell culture, 1 × 10^9^ ifu of NC-mimics lentivirus or 1 × 10^9^ ifu of miR-21-5p mimics lentivirus and 1 × 10^9^ ifu of NC-inhibitor lentivirus or 1 × 10^9^ ifu of miR-21-5p inhibitor lentivirus were respectively injected into the peritoneum. Four days post-injection, four mice from each group were sacrificed and peritoneal tissue samples were collected for further analysis. Sixteen mice were inoculated i.p. with BGC823-luc-D3 cells, and then bioluminescence images were captured after 2 weeks. All animal experiments were performed according to the guidelines of the Nanjing Medical University (NJMU) Institutional Animal Care and Use Committee.

### Statistical analysis

A TCGA miRNA expression dataset named TCGA-STAD/ Xena _M atrices/TCGA-STAD.mirna.tsv with version number 09-14-2017 was downloaded from the website of the UCSC cancer browser (http://xena.ucsc.edu/welcome-to-ucsc -xena/)^47^, containing 436 GC tissues and 41 normal tissues. All normalized expression values can be obtained from “TCGA-STAD.mirna.tsv” files. All statistical analyses were performed using GraphPad Prism software or Social Sciences (SPSS) software version 22.0 and displayed as means ± standard deviation (SD). Clinicopathological data were analyzed by using Pearson *χ*^2^ tests or Fisher’s exact test, and two-tailed unpaired Student’s *t*-tests were used to analyze differences between the two groups. For multiple comparisons, the results were corrected with the Bonferroni method. *P* *<* 0.05 was considered to be statistically significant (*P < 0.05, **P < 0.01, ***P < 0.001).
